# Supplementary material for: Content-rich biological network constructed by mining PubMed abstracts
Source: BMC Bioinformatics. 2004 Oct 8;5:147. doi: 10.1186/1471-2105-5-147 (PMC528731; doi:10.1186/1471-2105-5-147)
Supplement: Additional File 2 — The original results of the above study (non-essential files are deleted to keep the file size under the limit set by BMC bioinformatics). [file 1471-2105-5-147-S2.bz2 › chilibotAdditionalFile2/dip30/703ID10809785E18036/html/MALF_MALK.html]

 


 **MALF** and **MALK** 
  
Found 40 abstracts in PubMed, retrieved 30.  
 

 What does Google say? 
 PDF only 
| .edu only 

---

**Interactive relationship** (e.g. stimulation, inhibition, etc)

**Neutral relationship**- **MalK**  bound specifically to  **MalF**  and MalG and reconstituted a functional complex.  Ref: 9791180 Mol Microbiol, 1998
- Photolabeling of  **MalK**  required the presence of  **MalF**  but not MalG.  Ref: 8226895 J Biol Chem, 1993
- We focused on interactions between the nucleotide binding protein,  **MalK** , and the transmembrane proteins,  **MalF**  and MalG.  Ref: 9791180 Mol Microbiol, 1998
- 1998 Nature London 39 03 707 and are interpreted in favor of a role of the signature sequence in activating the hydrolyzing activity of  **MalK**  upon substrate initiated conformational changes in  **MalF**  MalG.  Ref: 10561582 Eur J Biochem, 1999
- Chemical cross linking detected specific interactions between  **MalF**  and MalG and between  **MalF**  and  **MalK** .  Ref: 2026607 J Biol Chem, 1991
- In addition, our results show that they play no role in the interactions with the other proteins of the maltose transport  **MalF** , MalG or  **MalK**  or chemotaxis Tar systems.  Ref: 2196376 J Mol Biol, 1990
- We used this approach and limited proteolysis with trypsin to show that binding and hydrolysis of ATP, inducing conformational changes in  **MalK** , modulate its interaction with  **MalF**  and MalG.  Ref: 9791180 Mol Microbiol, 1998
- This assay relies on the fact that, when a bifunctional  **MalK**  LacZ hybrid protein is associated with the  **MalF**  and MalG subunits, it is membrane bound.  Ref: 8226895 J Biol Chem, 1993
- We have used LacK as a tool to identify sites of interaction of  **MalK**  with the membrane integral components  **MalF**  and MalG.  Ref: 8951813 Mol Microbiol, 1996
- To this end, single cysteine residues were introduced into each subunit at several positions and the respective  **malF**  and malG alleles were individually co expressed with each of the  **malK**  alleles.  Ref: 10809785 J Biol Chem, 2000
- The MalG protein, together with  **MalF**  and  **MalK**  proteins, forms a multimeric complex in the membrane consisting of two  **MalK**  subunits for each  **MalF**  and MalG subunit.  Ref: 8437519 Mol Microbiol, 1993
- The maltose transport complex of Escherichia coli, a member of the ATP binding cassette ABC superfamily, is made up of two nucleotide binding subunits,  **MalK**  2, which hydrolyze ATP with positive cooperativity, and two transmembrane subunits,  **MalF**  and MalG.  Ref: 12093921 Proc Natl Acad Sci U S A, 2002
- These results indicate that a putative helical domain in  **MalK** , encompassing residues 89 140, is crucial for a functional, high affinity interaction with  **MalF**  and MalG.  Ref: 8951813 Mol Microbiol, 1996
- The results suggest that residues Ala 85, Lys 106, Val 114, and Val 117 in the helical segment of  **MalK** , to different extents, participate in constitution of asymmetric interaction sites with the EAA loops of  **MalF**  and MalG.  Ref: 10809785 J Biol Chem, 2000

**Non-interactive relationship** (e.g. studied together, co-existance, homology, etc.)

- In our studies of the oligomerization of this complex, we have shown that the ATP binding component,  **MalK** , forms a dimer in the absence of  **MalF**  and MalG.  Ref: 10037713 J Biol Chem, 1999
- The MTC is made up of two integral membrane proteins,  **MalF**  and MalG, and a peripheral membrane protein,  **MalK** .  Ref: 10037713 J Biol Chem, 1999
- The maltose transporter contains two transmembrane subunits,  **MalF**  and MalG, and two copies of a nucleotide hydrolyzing subunit,  **MalK** .  Ref: 11150310 J Biol Chem, 2001
- This uncoupled activity was not due to dissociation of the  **MalK**  ATPase subunit from the integral membrane protein  **MalF**  and MalG subunits.  Ref: 10648525 J Bacteriol, 2000
- First, we optimized the yield in purified complex protein by taking advantage of a newly constructed expression plasmid that carries the  **malK** ,  **malF**  and malG genes in tandem orientation.  Ref: 12225853 Biochim Biophys Acta, 2002
- The three membrane associated components of the transport system, the  **MalK** ,  **MalF** , and MalG proteins, have been solubilized from the membrane and maltose transport activity has been reconstituted in proteoliposome vesicles Davidson, A.  Ref: 2026607 J Biol Chem, 1991
- We found that when all three genes were present or when  **malF**  and  **malK**  were present together, the corresponding antigens were detected easily on Western immunoblots and were soluble in the non ionic detergent, Triton X 100.  Ref: 8226895 J Biol Chem, 1993
- The maltose transport system of E. coli is composed of a periplasmic maltose binding protein MBP, the presumed transmembrane channel made up of  **MalF**  and MalG proteins, and two copies of the ATPase subunit,  **MalK** .  Ref: 8206159 FEBS Lett, 1994
- We have investigated the proximity of residues in a conserved sequence EAA loop of  **MalF**  and MalG to residues in a helical segment of the  **MalK**  subunits by means of site directed chemical cross linking.  Ref: 10809785 J Biol Chem, 2000
- Analysis of flanking database sequences from M. tuberculosis and M. leprae revealed the presence of  **malF** , malG and  **malK**  like genes.  Ref: 10817516 Microbios, 2000
- In Escherichia coli, the binding protein dependent transport system for maltose and maltodextrins is composed of five proteins LamB, MalE,  **MalF** , MalG and  **MalK**  located in the three layers of the bacterial envelope.  Ref: 8437518 Mol Microbiol, 1993
- Each complex contains two  **MalK** , one  **MalF** , and one MalG proteins, providing two putative sites for ATP hydrolysis.  Ref: 2026607 J Biol Chem, 1991
- The products of the  **malF** , malG, and  **malK**  genes form a membrane associated complex that catalyzes the hydrolysis of ATP to provide energy for the transport event.  Ref: 1549599 Proc Natl Acad Sci U S A, 1992
- A putative helical domain in the  **MalK**  subunit of the ATP binding cassette transport system for maltose of Salmonella typhimurium MalFGK2 is crucial for interaction with  **MalF**  and MalG.  Ref: 8951813 Mol Microbiol, 1996
- Prior to measuring nucleotide binding, we examined membrane preparations for the presence of each subunit from strains that contained all possible permutations of the three structural genes,  **malF** , malG, and  **malK** .  Ref: 8226895 J Biol Chem, 1993
- Maltose negative mutants fall into two categories with respect to the cellular localization of the  **MalK**  ATPase in the first,  **MalK**  is membrane bound, as in wild type strains, while in the second, it is cytosolic, as in strains deleted in the  **malF**  and malG genes.  Ref: 9214624 EMBO J, 1997
- To study the mechanism of dominance we devised a means of measuring the ability of different wild type and mutant  **MalK**  proteins to interact with the  **MalF**  and MalG subunits.  Ref: 8226895 J Biol Chem, 1993
- The binding protein dependent maltose transport system of enterobacteria, a member of the ATP binding cassette ABC transporter superfamily, is composed of two integral membrane proteins,  **MalF**  and MalG, and two copies of an ATPase subunit,  **MalK** , which hydrolyze ATP, thus energizing the translocation process.  Ref: 10561582 Eur J Biochem, 1999
- ATPase activity was not due to dissociation of the  **MalK**  subunit from the integral membrane proteins  **MalF**  and MalG but could not be further stimulated by trehalose maltose binding protein TMBP, be it the native protein as isolated from T. litoralis or the soluble engineered protein.  Ref: 11453995 Eur J Biochem, 2001
- The complex, containing one copy each of the integral membrane proteins MalG and  **MalF**  and two copies of the peripheral cytoplasmic membrane protein  **MalK** , interacts with the periplasmic maltose binding protein to efficiently translocate maltose and maltodextrins across the bacterial cytoplasmic membrane.  Ref: 9573205 J Bacteriol, 1998
- The formation of a  **MalK**  dimer in the absence of  **MalF**  and MalG may represent an initial step in the assembly pathway of the MTC.  Ref: 10037713 J Biol Chem, 1999
- Cyclodextrin transport is mediated by the products of the cymE, cymF, cymG, cymD, and cymA genes, which are functionally homologous to the malE,  **malF** , malG,  **malK** , and lamB gene products of Escherichia coli.  Ref: 9573146 J Bacteriol, 1998
- In all experiments, the  **MalF** , MalG, and  **MalK**  proteins behaved as a multiprotein complex.  Ref: 2026607 J Biol Chem, 1991
- one important function of MBP is to transmit a transmembrane signal, through the membrane spanning  **MalF**  and MalG proteins, to the  **MalK**  protein on the other side of the membrane, so that ATP hydrolysis can occur.  Ref: 1549599 Proc Natl Acad Sci U S A, 1992
- Active transport of maltose in Escherichia coli requires the presence of both maltose binding protein MBP in the periplasm and a complex of  **MalF** , MalG, and  **MalK**  proteins FGK2 located in the cytoplasmic membrane.  Ref: 1406246 Mol Microbiol, 1992
- In addition to the water soluble maltose binding protein, the system comprises three membrane proteins,  **MalF** , MalG, and  **MalK** , which form a heterotetrameric complex FGK2 in the cytoplasmic membrane.  Ref: 8226895 J Biol Chem, 1993
- We report the characterization of TrmB, a protein of 3 00 apparent molecular weight, that is involved in the maltose specific regulation of a gene cluster in Thermococcus litoralis, malE  **malF**  malG orf trmB  **malK** , encoding a binding protein dependent ABC transporter for trehalose and maltose.  Ref: 12426307 J Biol Chem, 2003
- The proteins of this system are LamB in the outer membrane, maltose binding protein MBP in the periplasm, and the proteins of the inner membrane complex MalFGK2, composed of one  **MalF** , one MalG, and two  **MalK**  subunits.  Ref: 9401026 J Bacteriol, 1997
- We incubated purified  **MalK**  with inverted membrane vesicles containing  **MalF**  and MalG.  Ref: 9791180 Mol Microbiol, 1998
- The binding protein dependent maltose transport system of enterobacteria MalFGK 2, a member of the ATP binding cassette ABC transporter superfamily, is composed of two integral membrane proteins,  **MalF**  and MalG, and of two copies of an ATPase subunit,  **MalK** , which hydrolyze ATP, thus energizing the translocation process.  Ref: 10809785 J Biol Chem, 2000
